# Graphene coated magnetic nanoparticles facilitate the release of biofuels and oleochemicals from yeast cell factories

**DOI:** 10.1038/s41598-021-00189-7

**Published:** 2021-10-18

**Authors:** Santosh Pandit, Oliver Konzock, Kirsten Leistner, VRSS Mokkapati, Alessandra Merlo, Jie Sun, Ivan Mijakovic

**Affiliations:** 1 Department of Biology and Biological Engineering, Chalmers University of Technology, Kemivägen 10, 41296 Göteborg, Sweden; 2 The MathWorks AB, Regnbågsgatan 8B, 41755 Göteborg, Sweden; 3 Department of Microtechnology and Nanoscience, Chalmers University of Technology, Kemivägen 9, 41296 Göteborg, Sweden; 4 College of Physics and Information Engineering, Fuzhou University, Fuzhou, 350108 China; 5 Novo Nordisk FoundationCenter for Biosustainability, Technical University of Denmark, Kongens Lyngby, Denmark

**Keywords:** Biotechnology, Nanobiotechnology

## Abstract

Engineering of microbial cells to produce high value chemicals is rapidly advancing. Yeast, bacteria and microalgae are being used to produce high value chemicals by utilizing widely available carbon sources. However, current extraction processes of many high value products from these cells are time- and labor-consuming and require toxic chemicals. This makes the extraction processes detrimental to the environment and not economically feasible. Hence, there is a demand for the development of simple, effective, and environmentally friendly method for the extraction of high value chemicals from these cell factories. Herein, we hypothesized that atomically thin edges of graphene having ability to interact with hydrophobic materials, could be used to extract high value lipids from cell factories. To achieve this, array of axially oriented graphene was deposited on iron nanoparticles. These coated nanoparticles were used to facilitate the release of intracellular lipids from *Yarrowia lipolytica* cells. Our treatment process can be integrated with the growth procedure and achieved the release of 50% of total cellular lipids from *Y. lipolytica* cells. Based on this result, we propose that nanoparticles coated with axially oriented graphene could pave efficient, environmentally friendly, and cost-effective way to release intracellular lipids from yeast cell factories*.*

## Introduction

Microbial cell factories provide a sustainable source of biofuels and olechemicals^1^. Recently, environmental concerns regarding the use of fossil fuels has increased, due to their contribution to the greenhouse effect, acid rain, ozone depletion and climate change^2^. Hence, the interest towards the production and utilization of biofuel is growing. Different species of yeast, bacteria and microalgae are being genetically engineered to enhance the production of various biofuels and oleochemicals^2–5^. Lipids produced by these microorganisms can be used as an alternative for fossil fuels, both as a sustainable source of energy or chemicals otherwise derived from oil. This production is sustainable, because cheap and widely available carbon sources, even including a variety of industrial waste and biowaste, can be used to grow such microorganisms producing biofuels and oleochemicals^6,7^.

Yeast cell factories are particularly promising in this respect, due to their excellent ability to synthesize and accumulation of lipids^4^. *Yarrowia lipolytica* is one of the most promising yeast cell factories for production of oleochemicals and biofuels, because of its extremely high yield of lipids^4^, high levels of lipid accumulation^8^ and due to its metabolic diversity, which allows for production of a broad spectrum of unusual lipids^9^. However, *Y. lipolytica* and other cell factories do not actively secrete the produced lipids^10^. Instead, the lipids remain trapped inside the cells. Hence, different approaches for release of intracellular lipids from *Y. lipolytica* and other cell factories have been attempted, including microwave, ultrasound, thermal and mechanical treatments^11^, bio-based solvents^12^ and detergents^13^. However, most of these established methods are not selective for a specific product, they require high anounts of energy and require additional steps for lipid recovery^14^. Thus, these efforts failed in making biofuel and oleochemical production in yeasts and other cell factories economically feasible. The extraction of lipids from microbial cell factories remains a major bottleneck.

Nanosheets of graphene have been previously shown to interact with microbial membranes via computational simulations and experimentally^15,16^. The contact of graphene edges with microbial cell surfaces leads to penetration of the cell membrane and displacement of phospholipids onto the graphene surface^16–19^. In extreme cases, the interaction can lead to irreparable membrane damage and leakage of intracellular content^20^. The key parameter for effective membrane penetration, identified by our group and others, is the contact angle between graphene and the cell surface, which must be perpendicular to each other^18,21–25^.

In this study, we hypothesized that the exposed sharp edges of graphene spikes, deposited axially on magnetic nanoparticles, could interact with the membranes of yeast cells and thereby facilitate the release of valuable lipids (biofuels and oleochemicals) produced by these cell factories. To test this hypothesis, we decided to work with a strain of *Y. lipolytica* that has been genetically engineered for high-level production of lipids^26^. The interaction between graphene and membrane lipids is known to be mainly based on short-range van der Waals attractions^17^. These attractions promote the movement of lipid molecules from the biological membrane onto the graphene surface. For displacement of lipids from the membrane onto the graphene surface, the optimal contact angle between the graphene sheet and the biological membrane surface is 90º^15–18^.

Herein, we demonstrate a novel strategy to mechanically compromise the cell envelope in order to release intracellular lipids from *Y. lipolytica*. The treatment can be performed directly in the growth culture, without any interruption, and it can therefore be compatible with continuous release of intracellular lipids in fermenters*.* Our approach is based on metallic (iron oxide) nanoparticles coated with axially oriented graphene^27–29^, which show a considerable potential to release intracellularly accumulated lipids from yeast cell factories*.*

## Materials and methods

### Coating nanoparticles with graphene

Axial graphene coating was deposited on iron oxide (II, III) magnetic nanoparticles using PE-CVD, as described previously^24^. Briefly, iron oxide nanoparticles dispersed in water were drop-casted onto a SiO_2_ surface and heated at 80 °C for 20 min to remove water. After that, samples were kept at 350 °C for 10 min in the presence of H_2_ and Ar, and the temperature was increased to 450 °C for 10 min. The SiO_2_ surface with deposited nanoparticles was then loaded into a cold wall CVD system (Axitron, Black Magic). There the samples were heated rapidly (~ 300 °C/min) to the growth temperature of 775 °C and annealed in H_2_ and Ar atmosphere. A 75 W DC glow discharge plasma was ignited, and the graphene growth was initiated by introducing 15 sccm C_2_H_2_, 15 sccm H_2_ and 1000 sccm Ar. The density and orientation of coatings was confirmed by SEM (JEOL, JSM 6301F). The samples for SEM were sputter coated with gold (5 nm) before imaging. Raman spectra were measured by a WITec alpha300 R confocal Raman spectrometer equipped with a 100 × objective and spectrometer spectral range from 420 to 830 nm. Each spectrum was recorded in the range 500–3000 cm^−1^ with 10 min accumulation time and approximately 4 cm^−1^ resolution. The graphene-coated nanoparticles were detached from the SiO_2_ substrate carefully by using a surgical scalpel and used in all subsequent experiments.

### Lipid absorption by graphene nanoflakes and vertical coatings

Lipid absorption by different graphene coatings (monolayer parallel to the surface and PE-CVD-deposited perpendicular to the surface) was evaluated by contact angle measurements of lipid droplets on the coated surfaces. Commercially available lipids extracted from bacterial cells (Avanti Polar Lipids, Inc.) were used in these assays. The purchased lipid solution was diluted in 5% of dimethyl sulfoxide (DMSO). Droplets of DMSO (negative control) and lipids were placed onto graphene-coated surfaces and contact angle was measured for 1 h with an interval of 4 s. To evaluate the lipid absorption ability of graphene using X-Ray diffraction (XRD) and Fourier-transform infrared (FT-IR) spectroscopy, graphene nanoflakes in powder form (purchased from Graphitene) were used. The powder was ground before XRD measurements and mixed with lipids (or Mili-Q water), until visibly saturated. The X-Ray diffractograms were obtained using a Siemens Bruker D5000 powder diffractometer operating at 40 kV and 40 mA with nickel-filtered Cu Kα radiation (*λ* = 1.5418 Å) in the range 5° < 2*θ* < 70°. Bragg’s Law, *λ* = 2*d* sin(*Ɵ*), was used to calculate the distance *d* between adjacent graphene layers, where *λ* is the wavelength of the incident X-Ray beam and *Ɵ* is the diffraction angle. For FT-IR spectroscopy measurements, graphene powder was dispersed in lipids for 30 min, rinsed with water and dried. The absorption of lipid by graphene was examined by FT-IR. Infrared spectra of the samples were recorded using an attenuated total reflection (ATR) Alpha FT-IR spectrometer from Bruker, with a diamond crystal as refractive element, in the range 400 to 4000 cm^−1^ at a resolution of 2 cm^−1^.

### Interaction of vertical graphene coated nanoparticles with yeast cells

*Y. lipolytica* strain engineered for maximal production of lipids was grown in nitrogen restricted Delft medium for 5 days in a shake flask at 30 °C. The 5 day-culture (OD_600_ 35) were divided into three different fractions for further processing: a control fraction containing only *Y. lipolytica* culture, a fraction containing *Y. lipolytica* culture plus iron oxide nanoparticles and a fraction containing *Y. lipolytica* culture plus graphene-coated iron oxide nanoparticles. All samples were further incubated for 2 h with continuous agitation (1000 rpm) and then examined for release of intracellular lipids and morphological alterations. To examine the release of lipids, a fraction of treated cells was stained with 1 μL of BODIPY 493/503 (Thermo Fisher Scientific) solution (1 mg/mL in ethanol), which binds specifically to lipids and provides a green fluorescent signal. The stained yeast cultures were observed using a fluorescence microscope (Leica, DMI4000B). The acquired images were further processed to calculate the percentage of intracellular lipids (green stain appearing within cells) and released lipid droplets (stain appearing outside cells) by using Image J. The percentages of intracellular and released droplets were quantified by measuring the image area occupied by the droplets. The cumulative data represent quantification of intra- and extracellular lipid obtained by measuring the area of intracellular and extracellular lipid droplets in microscopy images with 200 cells, for both the control experiments (non-treated cells and cells treated with bare nanoparticles), and cells treated with graphene-coated nanoparticles. The morphological alterations of yeast cells were examined with SEM. A fraction of treated cells was fixed in 3% glutaraldehyde overnight. The fixed cells were dehydrated with graded series of ethanol (40%, 50%, 60%, 70%, 80%, 90% for 10 min and 100% for 15 min). The dehydrated cells were transferred to a clean cover glass and dried for 24 h at room temperature. The dried samples were sputter-coated with gold and examined by SEM (JEOL, JSM 6301F). Further, the effect of graphene coated nanoparticles on growth pattern of *Y. lipolytica* was evaluated. Graphene-coated nanoparticles was collected from two substrate plates (each having an area of 1 cm^2^) and dispersed in 1 ml of medium. This represents the standard working concentration of coated nanoparticles in all our experiments. Other concentrations were obtained by diluting the working concentration in culture medium. In order to evaluate the impact of nanoparticles on the growth profile, *Y. lipolytica* was grown overnight and inoculated in 150 µl of nitrogen limiting media with various concentrations of graphene coated nanoparticles to a starting OD_600_ of 0.05 in 96-well plates. The cells were cultivated at 30 °C and 200 rpm for 96 h. The growth was measured every 30 min by the Growth Profiler 960 (Enzyscreen B.V., Heemstede, The Netherlands).

## Results and discussion

**Figure 1:**
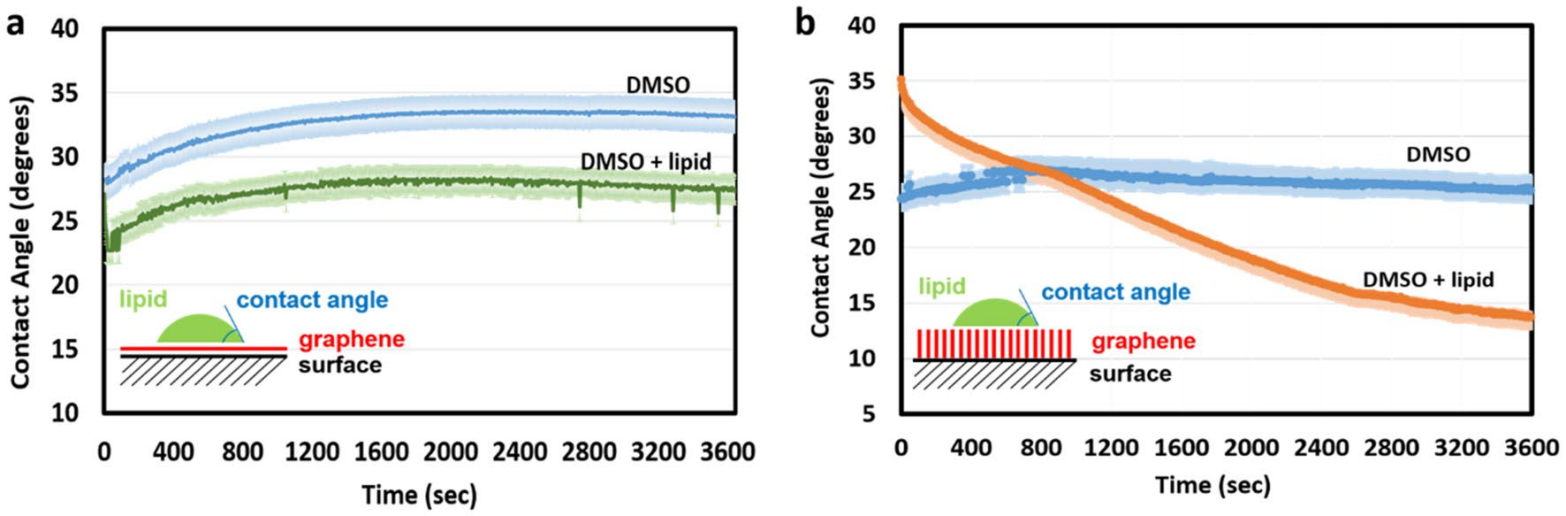
Time lapse measurements of contact angle of lipid droplets diluted in 5% dimethyl sulfoxide on (**a**) surface coated with monolayer graphene deposited parallel to the coated surface and (**b**) surface coated with vertically oriented graphene, perpendicular to the coated surface. 5% of dimethyl sulfoxide was used as a negative control.

The orientation of graphene has been demonstrated previously as a crucial parameter for the graphene-cell interaction, the extraction of phospholipids and release of intracellular materials^17,24^. Considering this phenomenon, initially we chose to compare the lipid absorption capacity of two types of differently oriented graphene coatings: a graphene monolayer parallel to the coated surface (Fig. 1a) and multiple layers of graphene stacked perpendicularly to the coated surface (Fig. 1b).

A droplet of lipid from biological membranes was deposited on both types of coated surfaces, and the contact angle between the surface and the droplet was measured over time. As shown in Fig. 1a, the contact angle of the lipid droplet deposited on a flat monolayer graphene coating remained constant throughout the experiment, indicating that a single layer of horizontal graphene cannot absorb significant amounts of lipid. By contrast, the contact angle of the lipid droplet deposited on vertically aligned graphene coating decreased progressively from 35º to less than 15º, within one hour (Fig. 1b). This result strongly suggested that a vertically oriented graphene coating, composed of many parallel layers of graphene, has excellent storage capacity for lipids derived from biological membranes.

**Figure 2:**
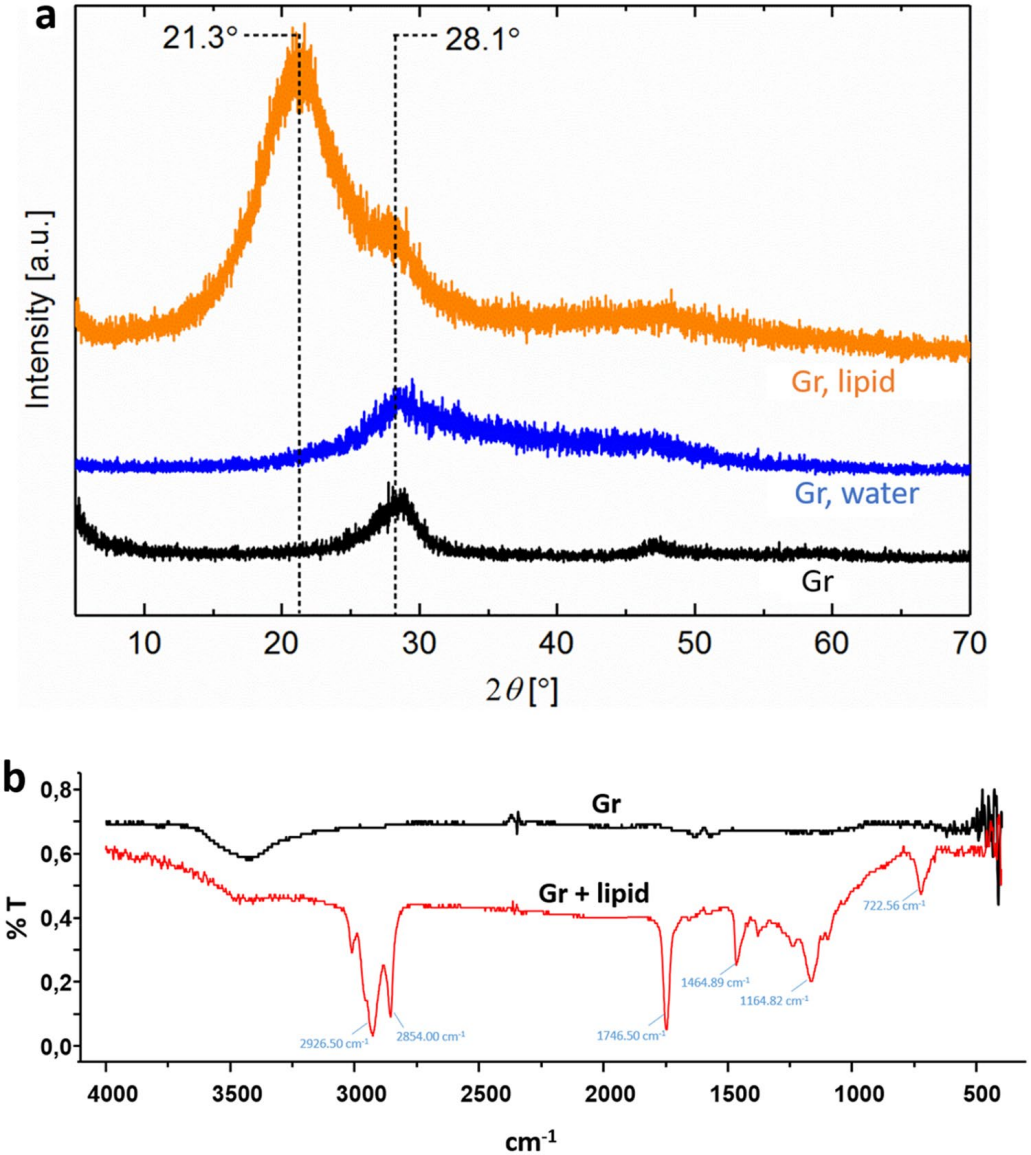
(**a**) X-ray diffractograms of graphene flakes alone (Gr), graphene flakes soaked in Milli-Q water (Gr + water) and graphene flakes soaked in lipids (Gr + lipid). (**b**) FTIR spectra of graphene flakes alone (Gr) and graphene flakes soaked in lipids (Gr + lipid).

Further, we speculated that this ability to absorb large amounts of lipids depends on intercalating lipid molecules between multiple layers of graphene in the coating. To examine this assumption, we next analyzed the interaction between multilayer graphene samples and biological lipids using X-ray diffraction (XRD) and Fourier-transform infrared (FT-IR) spectroscopy (Fig. 2). Figure 2a shows X-ray diffractograms obtained from multilayer graphene flakes alone and multilayer graphene flakes pre-soaked in either lipids or water. The main diffraction peak in the XRD pattern for untreated graphene was observed at 28.1°, which is characteristic of the (002) plane in graphene and graphite, as seen from comparison with the structural database entry JCPDS 75-1621. Bragg’s Law was applied to the (002) reflection pattern to calculate interplanar spacing distance, i.e., the distance between graphene layers, and was estimated at 0.32 nm for the untreated graphene. For the lipid-soaked sample, an additional peak was detected at 21.3°. The new peak corresponds to an interplanar spacing of 0.42 nm, a 31% increase from the layer distance in pure graphene.

This finding suggests that some fraction of the lipids is absorbed by graphene and inserted between the graphene layers. The unchanged peak position of the water-soaked sample in Fig. 2a suggests that the peak shift observed in lipid-soaked samples is not caused merely by a change in packing characteristics of the graphene flakes due to wetting with lipids. The XRD results indicated extra spacing between graphene layers upon interaction with lipids but did not actually prove the lipid absorption. To provide more direct evidence for lipid binding to multilayer graphene flakes, the flakes in powder form were mixed with biological lipids, washed and characterized using FT-IR spectroscopy (Fig. 2b). The FT-IR spectra of graphene mixed with lipids showed typical peaks related to biological lipids, suggesting the efficient absorption of lipids. Specifically, the characteristic peaks related to triglycerides, which are a major component of biological lipids, were observed in the recorded spectra: at 2926 cm^−1^ (C–H stretching (asymmetry)), 2854 cm^−1^ (C–H stretching (symmetry)), 1746 cm^-1^ (C=O stretching), 1465 cm^−1^ (C–H bending (scissoring)), 1165 cm^−1^ (C–O stretching and C–H bending), and 722 cm^−1^ (C–H bending)^30^. Taking together the findings from contact angle measurements (Fig. 1), XRD and FT-IR analysis (Fig. 2), we concluded that coatings based on multilayer graphene can soak up significant amounts of biological lipids. Next, we attempted to reproduce such coatings on the surface of spherical metallic nanoparticles.

**Figure 3:**
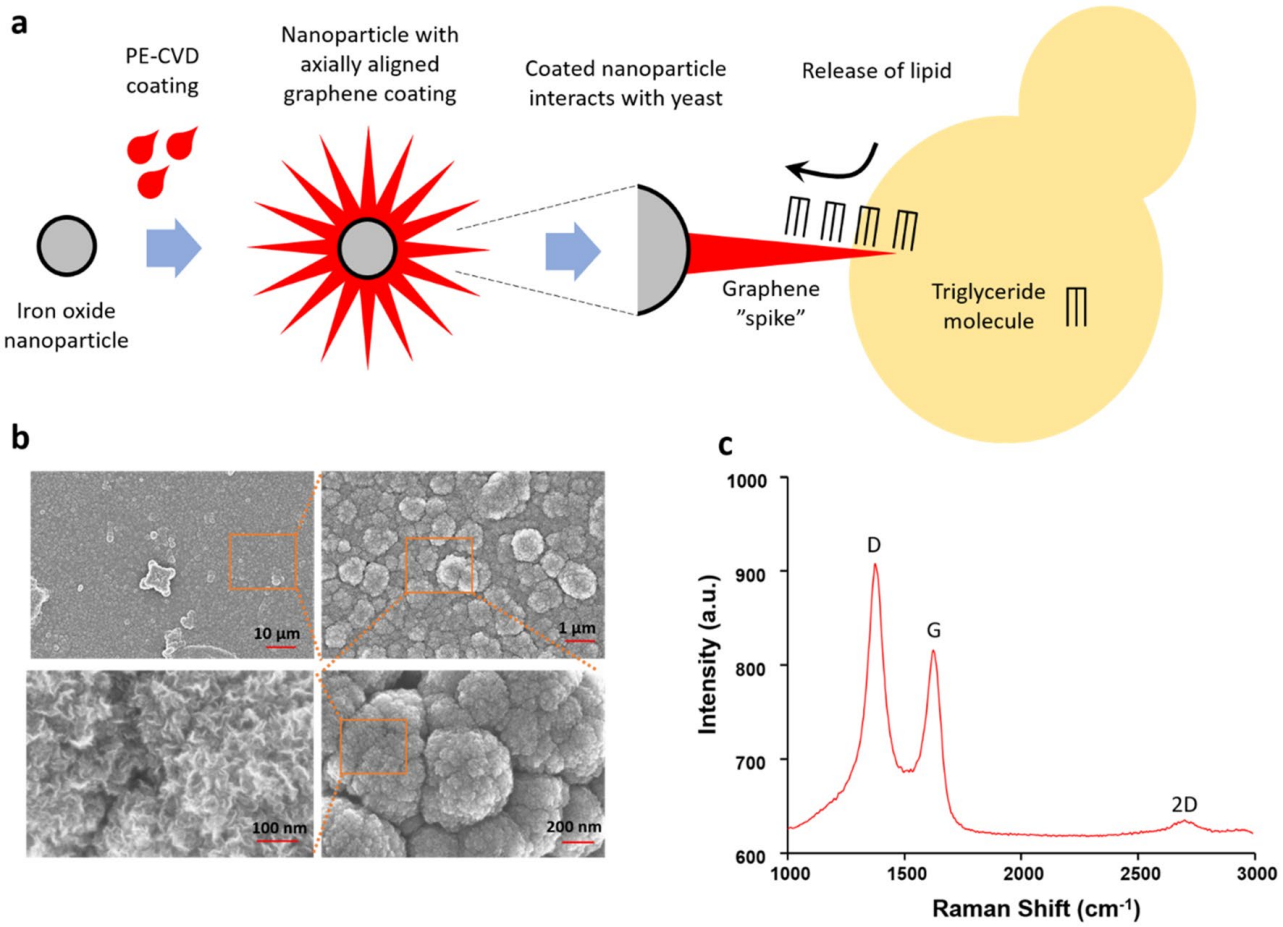
Characterization of iron oxide nanoparticles axially coated with PE-CVD graphene. (**a**) Overview of the coating procedure and lipid extraction strategy (prepared using Microsoft PowerPoint). Iron oxide nanoparticles were axially coated with PE-CVD graphene. Graphene spikes, in interaction with yeast cell, are expected to provoke extraction of lipids. In this schematic overview all constituents are not drawn to scale. (**b**) SEM images showing orientation and density of axial graphene coatings. Red lines indicate different levels of magnification. (**c**) Raman spectra (laser wavelength of 638 nm) of axial graphene coatings on iron oxide nanoparticles. The three main peaks characteristic for Raman spectra of graphene are marked: D, G, and 2D.

Iron oxide nanoparticles (average diameter of 25 nm) were axially coated with graphene, using the method of plasma-enhanced chemical vapor deposition (PE-CVD) (Fig. 3a). As a result, the surface of the iron oxide nanoparticles was completely covered with axially oriented graphene nano flakes, with height ranging from 60–100 nm (Fig. 3b). As shown in Fig. 3c, Raman spectroscopic examination of the coated nanoparticles revealed graphitic structure of the coatings, with relatively good quality. All Raman peaks specific for graphene, D, G and 2d, were detected^31^. The D peak (~ 1350 cm^−1^) being higher than the G peak (~ 1590 cm^−1^) is indicative of axial orientation and high density of flake boundaries. The low intensity of the 2D peak (~ 2650 cm^−1^) (intensity decreases with increasing number of graphene layers) indicated that the samples are a mixture of monolayer and multilayer graphene. Combining high magnification SEM imaging and Raman data, we concluded that the iron oxide nanoparticles were successfully axially coated with PE-CVD graphene, resulting in a complete and homogenous coating, with a large proportion of exposed sharp edges, containing on average < 10 layers of graphene.

Next, we asked whether these coated nanoparticles can effectively compromise the integrity of yeast cells to release intracellular lipids. Computational simulations of graphene interaction with cellular membranes describe the insertion of edges of free graphene nanosheets into the membrane, driven by strong van der Waals attractions and hydrophobic interactions with membrane lipids^17,32^. According to this mechanism, the graphene nanosheet cuts through the lipid membrane, with the edge of the nanosheet sinking into the lipid bilayer. Thereafter, the lipid extraction mode commences, where the nanosheet extracts the phospholipid molecules from the lipid bilayer onto its surface. This massive draining of lipids from the membrane onto the graphene sheet results in membrane deformation, and ultimately in loss of cell membrane integrity^17,32–34^. Following this extraction of phospholipids, strong hydrophobic interactions between the graphene and the lipids have been speculated to play an important role through nanoscale ‘dewetting’. This strong hydrophobic interaction can be compared to the hydrophobic collapse of many biomolecular self-assemblies, such as cell membrane formation and protein folding^35,36^. Hence, it has been suggested that nanoscale dewetting can provide the driving forces required for complete cellular collapse, leading to release of all intracellular content^37^. However, all available mechanistic simulations and experimental data showing this mechanism is based on the interaction of graphene with bacterial cell membranes. To the best of our knowledge, there are no reports showing the interaction of graphene nanosheets with yeast cells.

**Figure 4:**
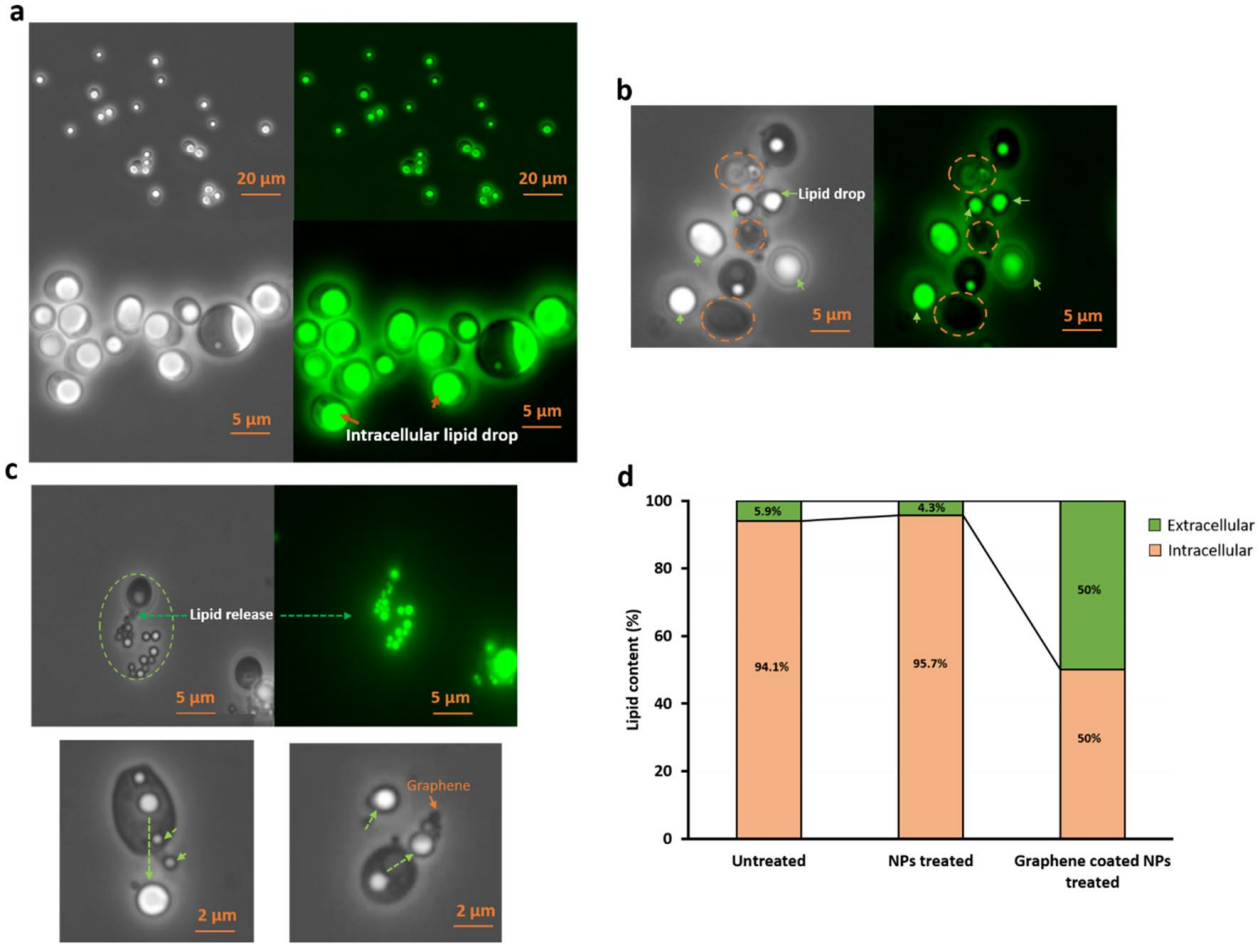
Bright field (left) and fluorescence (right) microscopic images of (**a**) untreated *Y. lipolytica* cells and (**b**) *Y. lipolytica* cells treated with graphene-coated nanoparticles. Samples were stained with BODIPY, which provides green fluorescence upon binding to lipids. The orange circles in the graphene-treated group show yeast cells with a complete release of intracellular lipid drops; green arrows are showing released lipid drops in the extracellular space. (**c**) Dropwise release of lipid droplets from yeast cells. The green circle is showing dropwise release (green arrow) and extracellular accumulation of lipid droplets. Zoomed-in images (bottom panels) show the interaction of graphene-coated nanoparticles and lipid drops, and their stepwise release from yeast cells. The green arrows show released lipid droplets and orange arrows indicate graphene-coated nanoparticles. (**d**) Quantification of intracellular and extracellular lipids, stained with BODIPY, in control and graphene-treated samples. Cumulative data obtained by examination of 200 untreated and 200 treated cells.

We therefore proceeded to test the ability of graphene-coated nanoparticles to extract lipids from yeast cells. In order to do so, fractions of *Y. lipolytica* culture grown for 5 days were treated with bare nanoparticles and graphene coated nanoparticles for 2 h with continuous agitation (1000 rpm). In order to assess if the release of lipid would be compatible with continuous growth in a fermentation process, no other treatment was applied. After 2 h of treatment with bare nanoparticles and graphene coated nanoparticles, yeast cells were examined by brightfield and fluorescent microscopy. The latter was combined with BODIPY staining (specific to lipid drops) that allows for visualization of lipids (including triglycerides), stained bright green (Fig. 4). All cells in the untreated control group were observed to contain large intracellular lipid droplets (Fig. 4a). By contrast, in all samples where cells were mixed with graphene-coated nanoparticles, extensive loss of intracellular lipids was observed. In some cases, the entire large lipid droplets moved out of the damaged cells (Fig. 4b). In other cases, lipids were more gradually drained from damaged cells, while interacting with graphene-coated nanoparticles.

**Figure 5:**
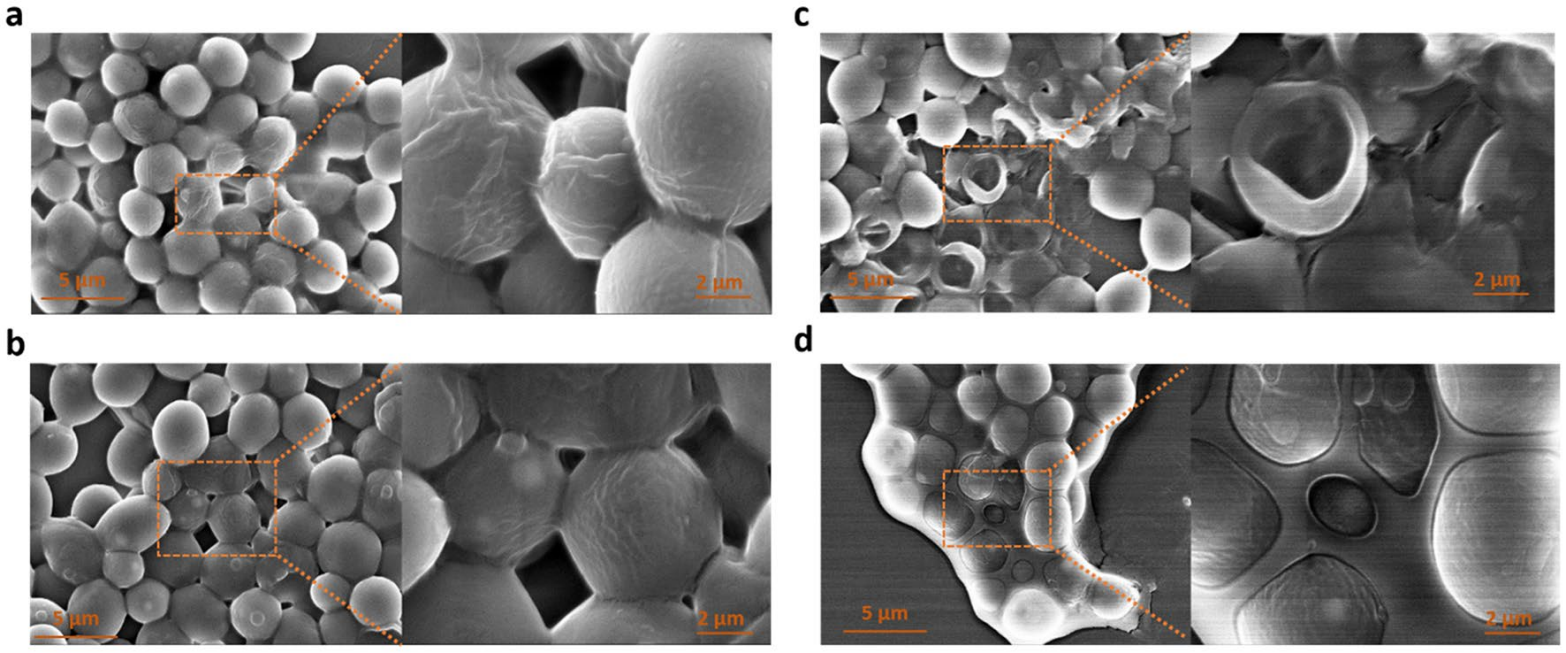
SEM images of *Y. lipolytica* cells (**a**) untreated, (**b**) treated with bare iron oxide nanoparticles, (**c**) treated with graphene-coated iron oxide nanoparticles. (**d**) Aggregation of *Y. lipolytica* cells on a glue-like matrix of extracted lipids.

A direct interaction between graphene-coated nanoparticles and smaller lipid droplets can be seen in Fig. 4c. All fluorescent microscopic images were further processed for quantification of total intracellular vs extracellular lipids (Fig. 4d). In the untreated group, only 5.9% of total lipid was found in the extracellular environment, due to the presence of some dead and disintegrated cells in the 5-day yeast culture. In samples treated with graphene-coated nanoparticles, about 50% of total lipid was detected outside the cells, suggesting a dramatic release of lipids from the *Y. lipolytica* strain. Finally, we asked whether the observed lipid release can be correlated exclusively to the presence of the axial graphene coating, or whether the iron oxide nanoparticles themselves have some capacity to damage yeast cells and release the intracellular lipids. To address this, cells treated with bare (non-coated) iron oxide nanoparticles were examined, and their lipid content was fully comparable to that of untreated cells, with only about 4.3% of total lipid present in the extracellular space. Thus, we concluded that the graphene coating is the key factor for release of lipids from cells. To more closely examine the morphology of *Y. lipolytica* cells exposed to graphene-coated nanoparticles, we further examined them by SEM (Fig. 5). The morphology of cells treated with bare iron oxide nanoparticles (Fig. 5b) was indistinguishable from cells that had received no treatment (Fig. 5a). This was consistent with fluorescent microscopy data (Fig. 4).

**Figure 6:**
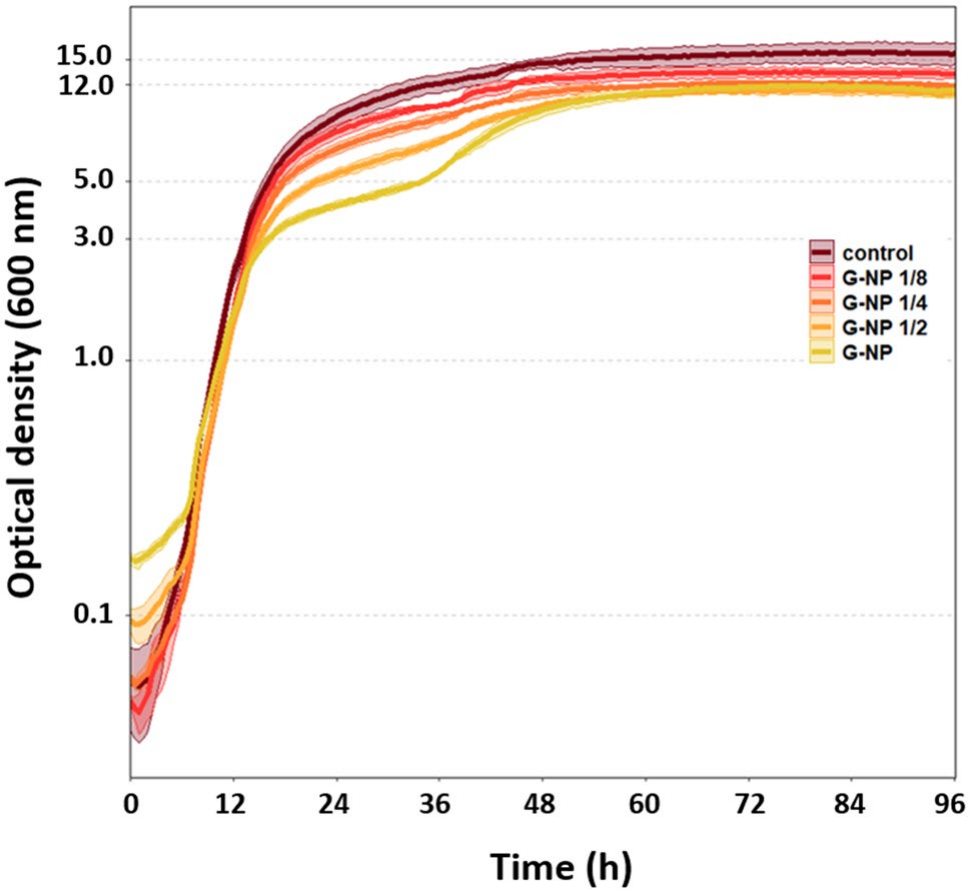
Growth of *Y. lipolytica* with and without presence of graphene- coated nanoparticles. Cells were incubated at 30 °C and 200 rpm and growth was monitored for 96 h at the interval of 30 min. Each curve and its respective shadow represent the mean values and standard deviation of biological triplicate experiments. G-NP denotes the standard working concentration of graphene-coated nanoparticles. G-NP 1/2, G-NP 1/4 and G-NP 1/8 are respective dilutions of the standard working concentration (G-NP).

By contrast, cells treated with graphene-coated nanoparticles exhibited various morphological defects (Fig. 5c), indicating extensive membrane damage and, in some cases, cell collapse. In the sample treated with graphene-coated nanoparticles, many *Y. lipolytica* cells were clustered, apparently held together by a lipid matrix presumably formed by lipids released from the damaged cells (Fig. 5d). Finally, the effect of graphene coated nanoparticles on growth pattern of *Y. lipolytica* was examined. As shown in Fig. 6, graphene-coated nanoparticles did not inhibit exponential growth of yeast. A weak concentration-dependent inhibition was observed at the entry of stationary phase, around OD_600_ = 3. However, the impact of nanoparticles on the final OD was minimal. This finding is very promising, since it indicates that lipid release from yeast cells can be achieved continuously without inhibiting growth, and therefore without compromising the metabolic yield of lipids. This technology could provide a significant improvement over existing methods to extract biofuels from microbial cell factories, thereby reducing energy consumption, extraction time and the use of hazardous chemicals. This encouraging finding is of course preliminary, and further optimization in scaled-up production setup should be performed in follow-up studies, to achieve an optimal balance between lipid production and lipid release.

## Conclusions

In conclusion, our results demonstrate that nanoparticles that are coated with sharp graphene spikes can be used to effectively break the cells and release intracellular lipids from *Y. lipolytica* (Fig. 4). Graphene coating is instrumental in piercing the yeast membrane (Fig. 5), and by itself, it is capable of storing large amounts of the extracted lipids (Figs. 1, 2). In our current experimental setup, a lot of lipid content ended up leaking directly to the extracellular environment, since the extent of cellular damage was quite severe. However, we have previously shown that it is possible to fine-tune the properties of vertical graphene coatings to make them less damaging to eukaryal cells^24^. Hence, we propose that our graphene-coated magnetic nanoparticles could be developed into a promising tool to break yeast cells to release intracellular lipid. The magnetic properties of the coated nanoparticles can be used to collect graphene a side while collecting the released lipid and could be compatible with an uninterrupted fermentation process. We propose that this simple technology could be used to resolve the major bottleneck for opening of yeast cells and release of intracellular lipid from yeast cell factories, thus paving the way for commercial and sustainable production of oleochemicals and biofuels. However, future studies should focus on finding the optimal size of graphene coating and concentration of coated nanoparticles which maximize the release of intracellular lipid while minimizing damage to yeast cells.
